# The Changing Spectrum of Surgically Treated Cystic Neoplasms of the Pancreas

**DOI:** 10.1155/2015/791704

**Published:** 2015-03-30

**Authors:** Jennifer K. Plichta, Jacqueline A. Brosius, Sam G. Pappas, Gerard J. Abood, Gerard V. Aranha

**Affiliations:** Department of Surgery, Loyola University Health System, Maywood, IL 60153, USA

## Abstract

*Introduction*. While the incidence of pancreatic cystic lesions has steadily increased, we sought to evaluate the changes in their surgical management. *Methods*. Patients with pancreatic cystic lesions who underwent surgical resection from 2003 to 2013 were identified. Clinicopathologic factors were analyzed and compared to a similar cohort from 1992 to 2002. *Results*. There were 134 patients with pancreatic cystic lesions who underwent surgical resection from 2003 to 2013, compared to 73 from 1992 to 2002. The most common preoperative imaging was a CT scan, although 66% underwent EUS and 63% underwent biopsy. Pathology included 18 serous, 47 mucinous, 11 pseudopapillary, and 58 intraductal papillary mucinous neoplasms (IPMN). In comparing cohorts, there were significantly fewer serous lesions and more IPMN. Postoperative complication rates were similar, and perioperative mortality rates were comparable. *Conclusion*. There has been a dramatic change in surgically treated pancreatic cystic tumors over the past two decades. Our data suggests that the incorporation of new imaging and diagnostic tests has led to greater detection of cystic tumors and a decreased rate of potentially unnecessary resections. Therefore, all patients with cystic pancreatic lesions should undergo a focused CT-pancreas, and an EUS biopsy should be considered, in order to best select those that would benefit from surgical resection.

## 1. Introduction

Neoplasms comprise 50% of cystic lesions of the pancreas [1]. They are divided into four main subtypes: serous cystic neoplasms (SCN), mucinous cystic neoplasms (MCN), solid pseudopapillary neoplasms (SPN), and intraductal papillary mucinous neoplasms (IPMN). Most frequently, these neoplasms are found incidentally, and the number of patients diagnosed with a neoplasm by imaging done for an unrelated reason is >2% [2]. The rise in routine use of computed tomography (CT) scans and magnetic resonance imaging (MRI) has led to an increase in the diagnosis of asymptomatic cystic neoplasms [3].

In general, IPMN, mucinous cysts, and SPN are removed surgically, given their significant potential for malignant transformation. Serous cystadenomas, in contrast, have a negligible rate of malignant transformation, although these lesions are more likely to be larger and cause symptoms due to mass effect [4]. It is considered standard of care to resect symptomatic serous cystadenomas that cause significant morbidity. However, the decision to resect asymptomatic masses has been more controversial, and management varies greatly by institution [4]. In the past, a more aggressive approach has been utilized for serous cystadenomas, due to concern for misdiagnosis based on standard, older imaging [4].

Pancreas-specific CT, developed in the mid-1990s, is a multidetector CT (MDCT) that allows for visualization of arterial, pancreatic, and portal venous phases and drastically enhances the diagnostic accuracy of pancreatic tumors [5]. The introduction of this technology along with endoscopic ultrasound- (EUS-) guided biopsies (fine needle aspiration FNA) has provided us with new ways to confirm pathology before operative intervention is undertaken. These advancements in imaging and technology have subsequently influenced the management of these neoplasms.

The objective of this study was to review our institution's experience with pancreatic cystic lesions and to characterize their presentation, diagnosis, and perioperative management. In order to determine how the spectrum of surgically treated cystic neoplasms of the pancreas may have changed over the years, we compared these patients to a previous study conducted by our institution from 1992 to 2002 [6].

## 2. Methods

Through a retrospective chart review, patients with pancreatic cystic lesions who underwent surgical resection from 2003 to 2013 at Loyola University Medical Center were identified (modern cohort). Patients with a prior history of pancreatic cancer were excluded. Clinicopathologic factors were assessed from the medical record, including clinical presentation, preoperative evaluation/imaging, surgeries performed, pathologic results, postoperative complications, and mortalities. The Social Security Death Index was utilized to determine current living status of patients in the modern cohort.

In the preoperative setting, a CT scan was typically the initial imaging performed. For patients with a contrast allergy or nonspecific findings on CT, an MRI was obtained. The main criterion for doing an EUS in the modern cohort was to get an accurate diagnosis of serous and mucinous tumors. If CT clearly showed a SPN or main duct IPMN, then EUS was not performed. However, EUS was done for asymptomatic branch duct IPMN to evaluate it for possible malignant features. In general, when an EUS was performed at our institution in the modern cohort, cyst fluid was sent for mucin, CEA (carcinoembryonic antigen), amylase, and cytology. Cysts that were high in amylase with no mucin or CEA and negative cytology were considered to be pseudocysts and were excluded. Fluids that had no mucin, low amylase, and low CEA were considered serous and were operated upon only if symptomatic. Cysts high in mucin with high CEA and atypical or malignant cytology went to surgery.

Select results were compared to a similar cohort of patients from 1992 to 2002 (data previously collected [6]). Statistical analyses were conducted using Stata 10.0 (StataCorp, College Station, TX). Categorical variables were analyzed using Chi-squared (*χ* ^2^) tests, and continuous variables were analyzed using Mann-Whitney *U* tests or Student's *t*-tests. Statistical significance was defined as *P* ≤ 0.05 (2-sided). This study was approved by the Loyola University Health Systems Institutional Review Board.

## 3. Results

**Figure 1:**
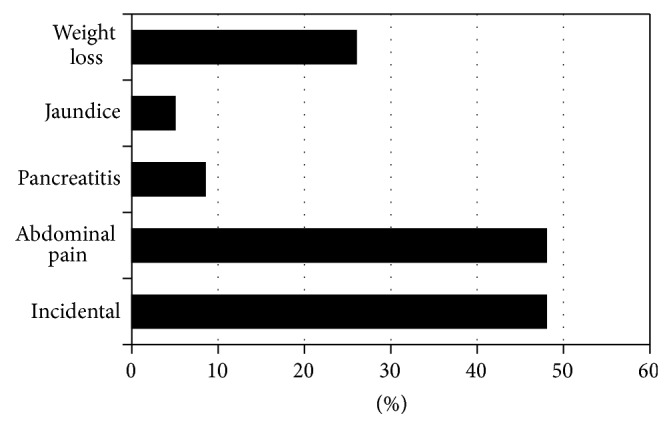
The most common clinical presentations in patients with pancreatic cystic lesions who underwent surgical resection from 2003 to 2013.

From 2003 to 2013, there were 134 patients with pancreatic cystic lesions who underwent surgical resection (modern cohort). The median age of the modern cohort was 66 years (range 18–88 years old). The historic cohort was comprised of patients who underwent surgical resection from 1992 to 2002 and included 73 patients. Patients were predominately females in both populations (67% versus 67%). The most common presenting symptom was abdominal pain (48%, *n* = 62), which was less common than observed in the historic cohort (64%, *P* = 0.013). However, the same number of lesions in the modern cohort was found incidentally on imaging (48%, *n* = 62). Other common clinical presentations for the modern cohort included 34 with weight loss, 11 with pancreatitis, and 7 with jaundice (Figure 1).

**Figure 2:**
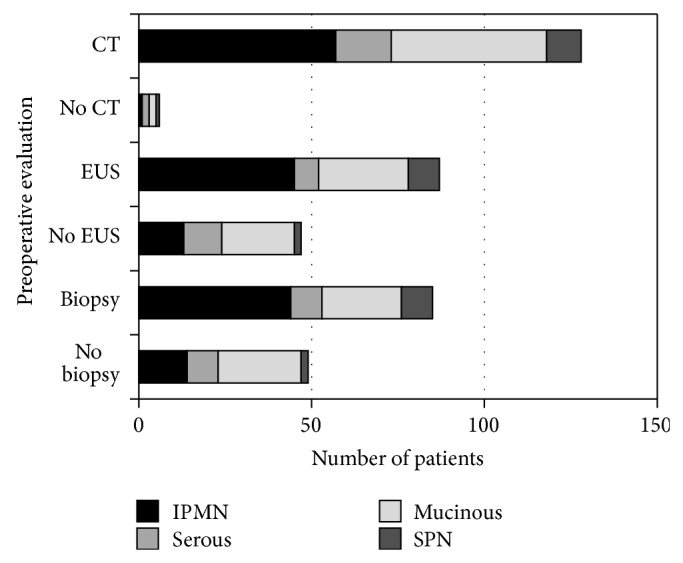
Preoperative evaluation of the modern cohort, including CT scans, endoscopic ultrasounds, and biopsy in select patients, stratified by the type of pancreatic cystic neoplasm. CT: computed tomography scan; EUS: endoscopic ultrasound.

**Figure 3:**
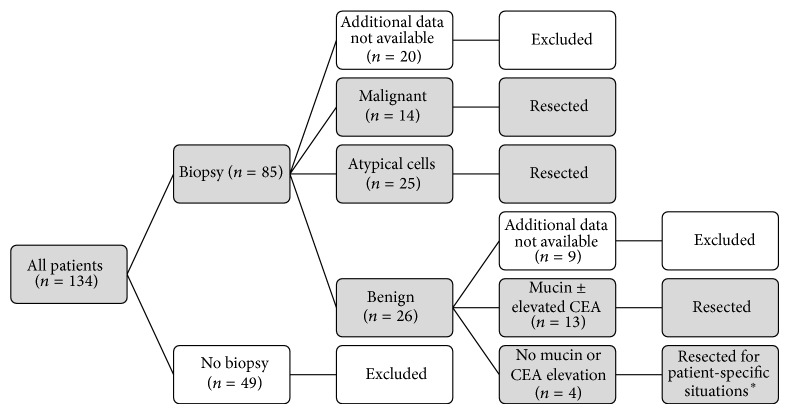
Results of preoperative biopsies and the algorithm for deciding when to operate. ^∗^The four patients resected who did not meet standard biopsy criteria were for (1) main duct IPMN; (2) elevated serum CA19-9; (3) two separate pancreatic tumors in a patient with Von Hippel-Lindau disease (one serous and one pancreatic neuroendocrine tumor); and (4) patient symptoms. IPMN: intraductal papillary mucinous neoplasms; CA: cancer antigen; CEA: carcinoembryonic antigen.

Prior to surgery, the majority of recent patients had a CT scan (98%, *n* = 128), while only 66% underwent EUS (*n* = 87) and 63% underwent biopsy (*n* = 85); the data is summarized in Figure 2 and is stratified by the type of cystic neoplasm. This data (preoperative imaging and biopsy) is not available in our records for the historic cohort, and we were unable to collect this data retrospectively. For the patients who underwent a biopsy (or fine needle aspiration), the indications for surgery included 14 patients with malignant lesions, 25 patients with atypical cells, 13 patients with mucin ±, an elevated CEA, and 4 patients with specific indications including (1) main duct IPMN; (2) an elevated CA19-9; (3) two separate pancreatic tumors in a patient with Von Hippel-Lindau disease (one serous, one pancreatic neuroendocrine tumor); and (4) patient symptoms. These results are outlined in Figure 3. The results of the biopsy were not available in 29 patients, as some patients were referred to our institution from an outside hospital and the records available were limited.

**Figure 4:**
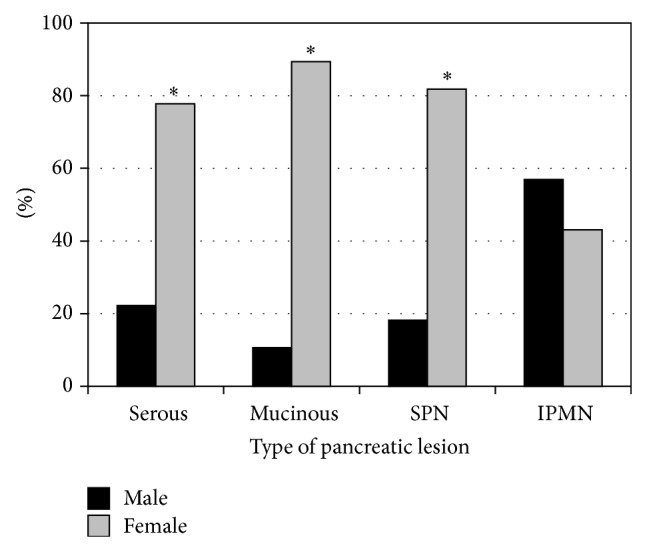
Comparison of pancreatic cystic lesions by gender in the modern cohort; ^∗^ *P* < 0.05. SPN: solid pseudopapillary neoplasms; IPMN: intraductal papillary mucinous neoplasms.

**Figure 5:**
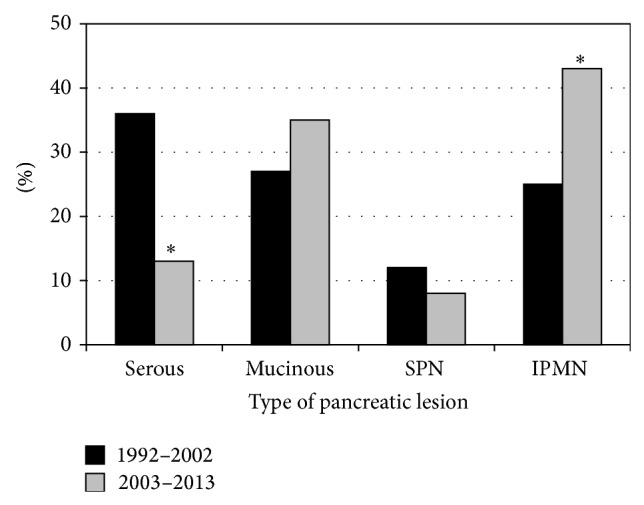
Comparison of pancreatic cystic lesions by patient cohort; ^∗^ *P* < 0.01. SPN: solid pseudopapillary neoplasms; IPMN: intraductal papillary mucinous neoplasms.

The main pathologic subtypes in the modern cohort included 18 serous lesions, 47 mucinous lesions, 11 SPN, and 58 IPMN. Most of the lesions were more common in females (serous *P* = 0.018, mucinous *P* = 0.000, and SPN *P* = 0.035), while IPMN lesions were similarly distributed (Figure 4). Malignancy was noted in 17% of the mucinous lesions and 38% of the IPMN. Compared to the historic cohort, there were significantly fewer serous lesions (13% versus 36%, *P* = 0.0002) and more IPMN (43% versus 25%, *P* = 0.008). There was no difference in the incidence of mucinous lesions or SPN (Figure 5).

**Figure 6:**
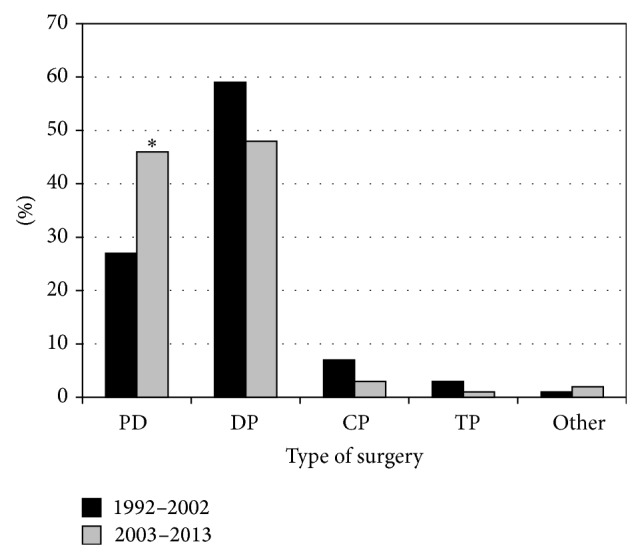
Comparison of surgeries performed by patient cohort; ^∗^ *P* < 0.01. PD: pancreaticoduodenectomy; DP: distal pancreatectomy; CP: central pancreatectomy; TP: total pancreatectomy.

**Figure 7:**
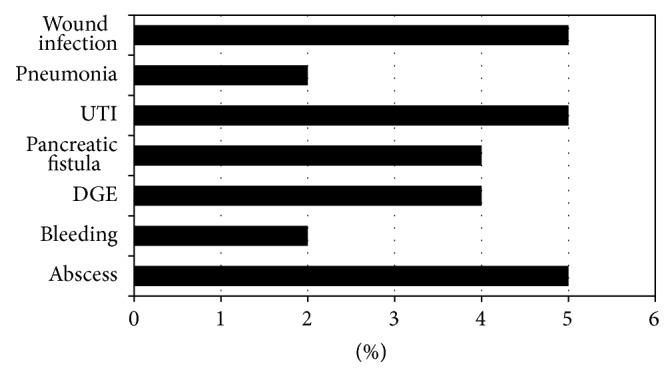
Most common postoperative complications. UTI: urinary tract infection; DGE: delayed gastric emptying.

**Figure 8:**
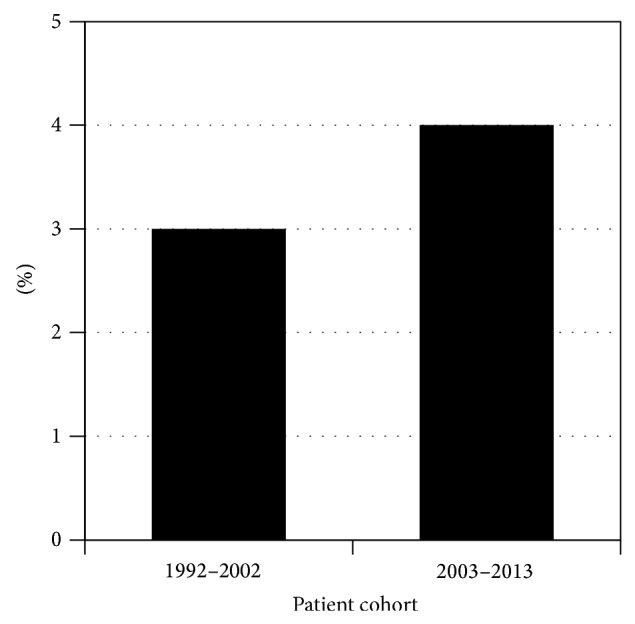
Comparison of mortalities by patient cohort.

The majority of patients in the modern cohort underwent pancreaticoduodenectomy (*n* = 62) or distal pancreatectomy (*n* = 64). In addition, there were 4 central pancreatectomies, 1 total pancreatectomy, and 3 other procedures (Table 1). In the historic cohort, the most common surgical resection was also the distal pancreatectomy (59%, *n* = 43); however, pancreaticoduodenectomies were significantly more common in the modern cohort (27% versus 46%, *P* = 0.008; Figure 6). Postoperatively, the most common complications in the modern cohort were wound infections (*n* = 7), intra-abdominal abscesses (*n* = 7), and urinary tract infections (*n* = 6). Other complications included 5 pancreatic fistulas, 5 with delayed gastric emptying, 3 gastrointestinal bleeds, and 2 pneumonias (Figure 7). Overall complication rates between the modern and historic cohorts were similar (32% versus 27%, *P* = 0.42), while pancreatic fistula rates were slightly less common in the modern cohort (4% versus 10%, *P* = 0.085). Perioperative mortality rates were comparable between both cohorts (3% versus 4%, *P* = 0.67; Figure 8).

## 4. Discussion

The increasing use of multidetector computed tomography (MDCT), magnetic resonance imaging (MRI), and endoscopic ultrasound (EUS) has specifically resulted in the increased recognition of pancreatic cystic lesions [7]. While some of these lesions are associated with malignancy, many of the lesions are asymptomatic at the time of diagnosis and benign in pathology. This has led to controversy in the surgical management of cystic pancreatic lesions [8]. Cystic lesions of the pancreas may be classified into neoplasms and pseudocysts [7]. The most common cystic neoplasms of the pancreas are serous cystic neoplasms (SCN), mucinous cystic neoplasms (MCN), solid pseudopapillary neoplasms (SPN), and intraductal papillary mucinous neoplasms (IPMN) [6].

We previously published our experience with the surgical management of cystic neoplasms between January 1992 and September 2002 [6]. In that decade, the most common cystic tumors surgically treated were SCN followed by MCN and both cystadenomas and cystadenocarcinomas, followed by IPMN, with SPN being the least common. Since that time, we and others [6, 9] have noted a change in the indications for surgery in pancreatic cystic neoplasms. At present, the most common indication for resection of a pancreatic cystic lesion is suspicion of malignancy, typically in IPMN and MCN. The prevalence of SCN has remained stable, while surgery for SCN has decreased dramatically. Why have all of these shifts occurred? The reasons are threefold: (1) SCN are often asymptomatic; (2) the incidence of malignancy in these tumors is quite low [10–13]; (3) the incidence of invasive carcinoma in IPMN is 15% at initial diagnosis and is cumulative over time [14]. Furthermore, improvements in MDCT, MRI, and EUS with cyst fluid analysis have helped differentiate the subtypes of cystic tumors prior to surgical treatment [15–17]. In a study by Lee et al., three radiologists were asked to interpret MDCTs and MRIs of 63 patients with cystic tumors of the pancreas, and the ability of each to predict malignancy was 77.8%, 73%, and 73%. These predictions were the same whether it was MDCT or MRI. The combination of MRI and MDCT was not significantly better but may merit future investigation [15].

The use of EUS in addition to CT (or MRI) has been recently evaluated by Khashab et al. [18]. This study evaluated 154 patients who underwent EUS and subsequent surgical resection and compared the diagnostic yield of the various imaging modalities. After CT or MRI, EUS increased the rate of correctly identifying neoplastic cysts by 36% and 54%, respectively, and thus appears to be a useful adjunct in the preoperative evaluation of patients with cystic neoplasms. In the modern cohort of our patients, there were slightly more patients with serous lesions that did not undergo preoperative evaluation with EUS, which may suggest that the addition of this study may have yielded a more accurate diagnosis preoperatively and possibly avoided surgical resection in select cases. Overall, the decreased rate of surgical resection for serous lesions is likely related to the increased use of CT scans and confirmation by EUS when necessary.

Cyst fluid analysis from EUS has also demonstrated utility in diagnosing SCN [13, 19, 20]. In one study at Indiana University Medical Center, analysis of the cyst fluid using VEGF-A correlated with pathologic diagnosis [13]. For SCN specifically, a high level of VEGF-A (8500 pg/mL) had a sensitivity of 100% and specificity of 97%. With a cutoff of 200 pg/mL, VEGF-C identified SCN with 100% sensitivity and 90% specificity. Another study from Massachusetts General Hospital demonstrated a low CEA level and a low amylase level in SCN lesions [19]. Therefore, the surgical intervention in SCN today is limited to those patients who are symptomatic, those who have large tumors, and those who have enlarging lesions. Our policy on asymptomatic SCN is to get a MDCT at six-month intervals for two years and then yearly for a period of five years.

Currently, the major controversy surrounding cystic tumors of the pancreas is related to IPMN. IPMN lesions can be classified into 3 subtypes: (1) main duct IPMN (MD-IPMN); (2) mixed duct IPMN; and (3) branch duct IPMN (BD-IPMN). In a review of a published series of 50 or more cases, the frequency of malignancy was 61.6% and the frequency of invasive IPMN was 43.1%. Considering these incidences, it was felt that surgical resections were strongly recommended in all fit patients in whom the main duct was dilated greater than 5 mm in diameter and in those who presented with symptoms of jaundice, pancreatitis, diabetes, and/or steatorrhea [21]. However, much of the controversy surrounds the decision to resect or observe BD-IPMN lesions. Tanaka et al. [21] published international consensus guidelines in 2012 for the management of IPMN of the pancreas. The frequency of malignancy in resected BD-IPMN was 25.5% (range 6.3–46.5%); the frequency of invasive cancer was 17.7% (range 1.4–36.7%). This may warrant consideration of resection in many patients; however, BD-IPMN are often discovered incidentally [8] and occur in elderly patients who may have comorbid conditions. Therefore, in these patients, surgery may be indicated when the cyst is greater than 3 cm, imaging follow-up reveals growth, a mural nodule is present, and/or positive cytology is obtained [21]. In the absence of these clinical indications, the consensus group concluded that BD-IPMN lesions less than 3 cm could be observed, particularly in elderly patients. In comparison, other authors have recommended consideration of enucleation for BD-IPMN [22]. However, the incidence of pancreatic fistula was 43%, and, as such, we have not undertaken enucleation for BD-IPMN. In reviewing our own data (reported previously [23]), preoperative testing did not reliably differentiate main versus branch duct lesions, and thus the decision to observe should be undertaken cautiously. Nevertheless, there does appear to be a role for observation in patients with lesions that are asymptomatic and not showing growth with no mural nodules.

Tanaka et al. also published guidelines for MCN. MCN is defined by the presence of ovarian stroma and has a low prevalence of invasive carcinoma [21]. These tumors often occur in young women, and the risk of progression to invasion, though not accurately known, is possible [24, 25]. Based on this, the consensus in MCN is to resect, which can often be achieved laparoscopically when involving the body of the pancreas [21]. In the frail elderly patient, ablation may be considered [21]. Because of the potential for surgical management, it is important to distinguish mucinous from nonmucinous cystic neoplasms in the preoperative evaluation. The utility of EUS cytology and cyst fluid analysis (CEA and amylase) has previously been assessed and proven to be highly specific for differentiating these tumors, while EUS morphology alone did not distinguish between the two groups [20].

SPN of the pancreas is an entity that occurs mainly in young women in the third decade of life; however, as in our series, others have seen a few cases which occur in men [26, 27]. These tumors have both solid and cystic elements and can present with solitary lesions in the head, body, and tail of the pancreas, and a certain percentage of patients will present with metastases at the time of diagnoses [26, 27]. Because these lesions occur mainly in young women and have a malignant potential, the general consensus is to resect, depending on the location of the tumor, either by pancreaticoduodenectomy or distal pancreatectomy ± splenectomy. Cure rates are high, and patients often do well after removal of metastases when they occur in the liver [26, 27].

Although our work represents an update on the management of pancreatic cystic neoplasms, it has several limitations. It is a retrospective review of data from one institution and thus has an inevitable selection bias. The ideal study would include data on those patients with pancreatic cystic lesions who do not undergo surgery, which could then serve as a population for comparison. Patients managed nonoperatively, however, are followed up by several types of physicians, often including nonsurgeons, which makes identification of such patients difficult. In addition, some are only followed up for a few years and are then released from follow-up. Because of these pitfalls, the overall incidence of pancreatic cystic neoplasms could not be calculated in our study. However, there were approximately 75 patients followed up by surgeons during the second era, based on CT and EUS criteria; in the first era, we operated on all cystic lesions that were proven not to be pseudocysts and were evaluated by a surgeon. The majority of these were serous cystic adenomas, and they are followed up with CT scans every six months for two years and yearly scans for three years. Only one branch duct IPMN became symptomatic during this time period and required surgical intervention.

## 5. Conclusions

In summary, our experience with cystic tumors of the pancreas in the modern era reveals a dramatic change in the indication for surgery, which is mainly related to SCN and IPMN. In the first decade reviewed, SCN was the most commonly resected cystic tumor, and, in the second decade, IPMN was the most commonly resected tumor and SCN was the second least. This is likely related to the advancements in imaging and fluid analysis using EUS. Our present approach to SCN is to surgically resect those that are symptomatic, those that are large, and those that are growing on sequential follow-up imaging. Because of the possibility of a benign MCN undergoing malignant transformation, we recommend resection of these tumors. SPN lesions often occur in young women, can harbor malignancy, and sometimes present with metastases. Therefore, we suggest that resection is indicated in relation to the site of the tumor in the pancreas. These patients may also benefit from metastasectomy. MD-IPMN patients who present with jaundice, pancreatitis, steatorrhea, diabetes, and/or main duct diameter greater than 5 mm should undergo surgical resection. This may include pancreaticoduodenectomy or a total pancreatectomy. In BD-IPMN, surgery is reserved for those who are symptomatic, those with enlarging lesions on follow-up imaging, those whose cytology reveals atypia, and those with a mural nodule on imaging. For those with mixed type IPMN, surveillance is performed for high risk patients with comorbidities and those who have a borderline dilated duct that is less than 5 mm. However, in those patients who have major pancreatic duct dilatation and have cytology that is atypical, we recommend resection. It is our contention that the proper use of imaging and cyst fluid analysis has led to less surgery in asymptomatic SCN and increased recognition of IPMN where the prevalence of malignancy is a reality.

## Figures and Tables

**Table 1:** Types of surgeries performed based upon the pathology of the pancreatic cystic lesions.

Type of tumor	PD	DP	CP	TP	Other
SCN	9	8	0	0	1
Mucinous (MCN)	17	26	1	1	2
Cystadenoma	12	23	1	1	2
Cystadenocarcinoma	5	3	0	0	0
SPN	3	8	0	0	0
IPMN	33	22	3	0	0

Total	62	64	4	1	3

SCN: serous cystic neoplasms; MCN: mucinous cystic neoplasms; SPN: solid pseudopapillary neoplasms; IPMN: intraductal papillary mucinous neoplasms; PD: pancreaticoduodenectomy; DP: distal pancreatectomy; CP: central pancreatectomy; TP: total pancreatectomy.
